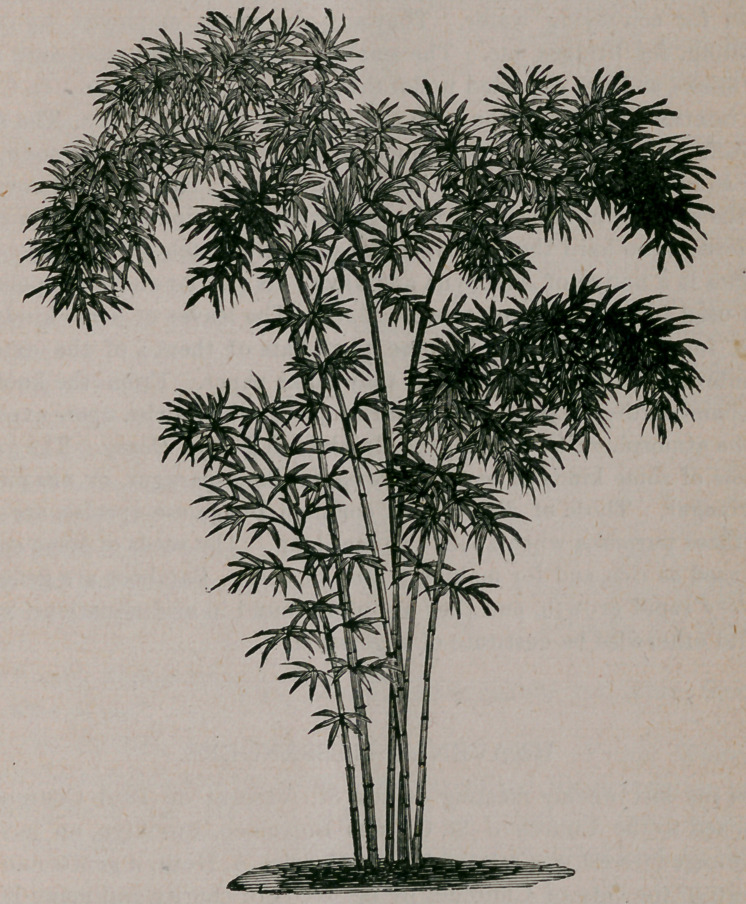# The Bamboo

**Published:** 1888-04

**Authors:** 


### THE BAMBOO.

This is a genus of grasses, of which most of the species attain a great size, many of them twenty or thirty feet, some seventy or one hundred feet in height. The species are numerous, and are found in tropical and subtropical regions, both of the eastern and western hemispheres. They are of great importance to the inhabitants of the countries in which they grow. All of them have a jointed subterranean root-stock which throws up stems from ten to one huudred feet high. These are generally straight and erect; although one large species (*B. agrestis*), common in dry mountains situations in the southeast of Asia, has crooked, and somtimes, creeping stems. The stems grow to their full height unbranched, but afterwards throw out straight horizontal branches, especially in their upper parts, forming a dense thicket. Some of the smaller kinds are often planted as hedges. The stems are jointed like those of other grasses, very hard, but light and elastic, hollow, containing only a light spongy pith, except at the joints or nodes, where they are divided by strong partitions. They are, therefore, readily converted into water vessels of various sorts ; and when the partitions are removed they are used as pipes for conveying water. They are also much employed for house building, for bridges, etc. The smaller stems are converted into walking sticks, and are exported under the name of bamboo cane. In China, the interior portions of the stem are used for making paper. The stems of different species vary very much in the thickness of the woody part, and so in their adaptation to different purposes. The external covering of the stem is, in all the species, remarkably silicious ; the stem of *B. tabacaria* is so hard that it strikes fire when the hatchet is applied. This species is a native of Amboyna and Java ; its slender stems are polished, and used for the stalks of tobacco pipes. The leaves of some kinds are used for thatch, and the Chinese plait hats of them ; of the external membrane of the stems of some they make paper. From the knots of the bamboo there exudes a saccharine juice, which dries upon exposure to the atmosphere, and which the Greeks call *Indian Honey*. The young shoots of some kinds of bamboo are eaten like asparagus, or are pickled in vinegar. Those of *B. Tulda*, a common Bengalese species, are used for these purposes when about two feet long. The seeds of some species are used as rice, and for making a kind of beer. Bamboos are generally of very rapid growth, and they are often found in arid situations, which would otherwise be destitute of vegetation.

## Figures and Tables

**Figure f1:**